# Assessment of Physicians’ Knowledge and Practices Regarding Cervical Cancer Screening in University and Non-University Hospitals in Northeastern Democratic Republic of the Congo

**DOI:** 10.3390/healthcare14213594

**Published:** 2026-10-23

**Authors:** Yvette Neema Ufoy Mungu, Burubu Lisi-Ankiene Junior, Elettra Bianchi, Joëlle Desreux, Katenga Bosunga, Vincent Bours, Roland Marini Djang’eing’a, Anne-Marie Etienne

**Affiliations:** 1 Department of Gynecology and Obstetrics, Faculty of Medicine, University of Kisangani, Kisangani P.O. Box 2012, Democratic Republic of the Congo; katengabosunga@gmail.com; 2 Department of Biomedical and Preclinical Sciences, GIGA Cancer—Human Genetics, Faculty of Medicine, University of Liege, 4000 Liege, Belgium; vbours@uliege.be; 3 Department of Gynecology and Obstetrics, Faculty of Medicine, University of Kikwit, Kikwit P.O. Box 02202, Democratic Republic of the Congo; burubujunior@gmail.com; 4 Department of Anatomopathology, University Hospital of Liege, 4000 Liege, Belgium; ebianchi@chuliege.be; 5 Department of Gynecology, Faculty of Medicine, University of Liege, 4000 Liege, Belgium; joelle.desreux@citadelle.be; 6 Laboratory of Analytical Pharmaceutical Chemistry, Center for Interdisciplinary Research on Medicines, Faculty of Medicine, University of Liege, 4000 Liege, Belgium; rmarini@uliege.be; 7 Department of Galenic Pharmacy and Drug Analyses, Faculty of Pharmaceutical Sciences, University of Kisangani, Kisangani P.O. Box 2012, Democratic Republic of the Congo; 8 Department of Psychology, Health Psychology, Faculty of Psychology, Speech Therapy and Educational Sciences, University of Liege, 4000 Liege, Belgium; am.etienne@uliege.be; 9 Research Unit for a Life-Course Perspective on Health and Education, University of Liege, 4000 Liege, Belgium

**Keywords:** cervical cancer, screening, knowledge, self-reported practices, Democratic Republic of the Congo

## Abstract

**Background/Objectives:** In the Democratic Republic of the Congo (DRC), cervical cancer (CC) is the most common gynecological cancer. This study assesses physicians’ knowledge and self-reported clinical practices regarding CC screening in both university and non-university hospitals across four provinces in northeastern DRC. **Methods:** This cross-sectional study was conducted from 15 April 2025 to 15 June 2025, in Kisangani, Buta, Isiro, and Bunia, the respective capitals of the provinces of Tshopo, Bas-Uele, Haut-Uele, and Ituri in the DRC. We collected data using a self-administered questionnaire completed by 26 physicians from university hospitals and 106 physicians from non-university hospitals. Data analysis was performed using R software, version 4.6.0. **Results:** Of the 175 eligible physicians, 132 (75.4%) were surveyed. Respondents demonstrated similar knowledge regarding most screening modalities regardless of hospital type. However, physicians at university hospitals had significantly better knowledge of the target age range (*p*-value = 0.001) and diagnostic confirmation test (*p*-value = 0.03). Overall, 34 respondents (25.8%) had sufficient knowledge, with higher proportions among those at university hospitals and those residing in Bunia, Ituri Province (*p*-value = 0.001 and *p*-value = 0.02, respectively). Regarding self-reported practices, 57.7% of physicians in university hospitals and 49.0% of those in non-university hospitals reported screening 1–24 women in the previous 12 months, with naked-eye VILI use reported more frequently (57.7% vs. 32.1%, *p*-value = 0.03). Only six participants (4.5%) reported a high level of screening- and management-related activities, including some obstetrician–gynecologists (*p*-value = 0.02). **Conclusions:** Our findings revealed knowledge gaps and suboptimal self-reported CC screening practices among physicians in northeastern DRC, underscoring the need for strengthened training and professional support. Greater involvement of obstetrician–gynecologists, mentorship from university hospitals, and collaboration across provinces may help improve CC screening.

## 1. Introduction

Cervical cancer (CC) is the fourth most common cause of cancer death among women worldwide, with an estimated 660,000 new cases and approximately 350,000 deaths in 2022 [1]. In the Democratic Republic of the Congo (DRC), CC is the most commonly diagnosed cancer and the leading cause of cancer-related death among women [1,2]. In 2020, it was the second most common gynecological cancer among women aged 15–44 years [2]. According to the International Agency for Research on Cancer (IARC), an estimated 8705 new cases of CC and 6187 deaths from the disease occurred in the DRC in 2022 [3].

This cancer is caused by persistent cervical infection with oncogenic types of human papillomavirus (HPV), which are transmitted sexually. While approximately 90% of infections are cleared spontaneously within 12 to 18 months, persistent infection may lead to cervical precancerous lesions and cancer over 10 to 20 years. This long progression period provides an opportunity for screening, early detection, and effective treatment [4,5].

In 2020, the World Health Organization (WHO) developed a strategy to eliminate CC as a public health problem by 2030 based on three pillars: vaccination against HPV, screening, and effective treatment of precancerous lesions and CC [6]. Since 2021, the WHO has recommended HPV as the primary screening method, instead of traditional cytology. However, in low- and middle-income countries (LMICs), screening using visual tests (notably, visual inspection with acetic acid (VIA) and visual inspection with Lugol’s iodine (VILI)) is still permitted, as part of the “screen-and-treat” approach [7,8]. Although women living in LMICs account for 85–90% of new CC cases and related deaths, access to vaccination, screening, and treatment remains limited in these settings [9,10].

In Central Africa, CC screening data are available in only a few countries. In the DRC, screening is only carried out occasionally, mainly on the initiative of a few organizations such as the Congolese Society of Gynecology and Obstetrics [11], or within the framework of pilot projects implemented in some selected cities [12,13].

Healthcare facilities in the DRC are organized according to a two-tiered pyramid hierarchy. The first tier comprises health centers that provide basic preventive and curative care. The second tier is subdivided into three levels: (i) general referral hospitals (GRHs), (ii) provincial hospitals, and (iii) university hospitals [11,14]. According to the 2015 national strategy to combat gynecological cancers in the DRC, GRHs should be able to diagnose using visual methods during colposcopy, cytology, and biopsy. Conization and cryotherapy should be available for treating precancerous lesions in these healthcare facilities. In addition, university hospitals should have molecular biology laboratories for HPV testing, as well as other effective therapeutic and diagnostic tools [12].

Almost ten years after the strategy was developed, its implementation has been only partially evaluated. However, many studies have reported low screening coverage in LMICs, mainly due to poor compliance among women, but also due to a lack of skills among healthcare professionals and limited hospital equipment [15,16,17].

The DRC suffers from insufficient healthcare coverage, both in terms of qualified personnel and infrastructure, with an average of only 1 to 1.2 doctors per 1000 inhabitants [18]. The provinces of Tshopo, Ituri, Haut-Uele, and Bas-Uele are particularly affected by these shortages and collectively cover approximately 22% of the country’s national territory [19]. Due to insecurity and intensive mineral exploitation, these provinces also report the highest Human Immunodeficiency Virus (HIV) prevalence rates (over 4% vs. 1.2% nationwide) [20].

Since HIV accelerates the progression of HPV infection to cancer [7,21] and considering the size of the affected area, it is essential that physicians practicing in these regions possess adequate knowledge and skills to screen for CC. Accordingly, our study aims to assess physicians’ knowledge and self-reported clinical practices regarding CC screening among physicians practicing in university and non-university hospitals in the capital cities of four provinces in the northeastern DRC, and to identify the factors associated with these findings.

## 2. Materials and Methods

### 2.1. Study Design and Setting

**Figure 1:**
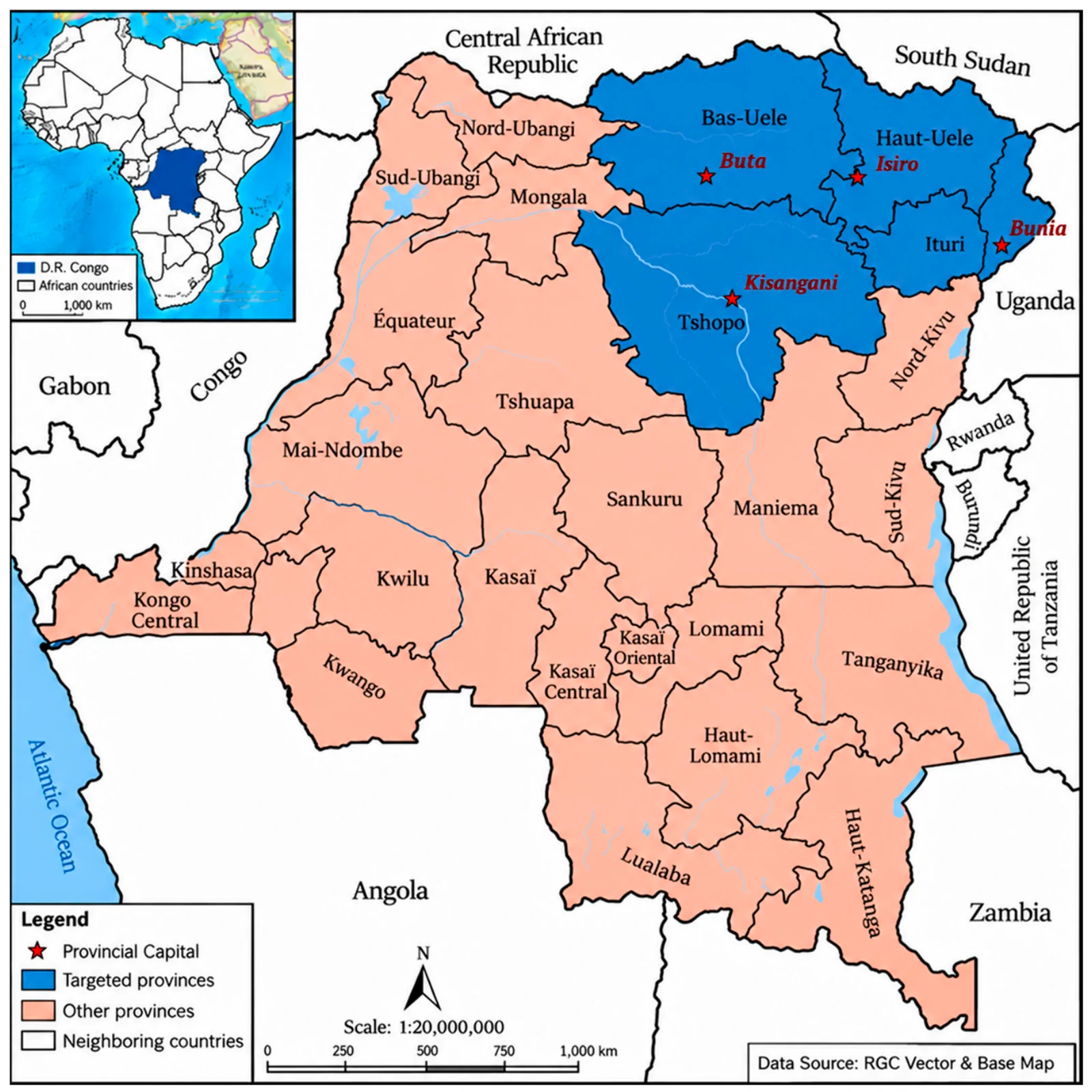
Geographical location of the cities of Kisangani, Bunia, Buta and Isiro/DRC (map created using QGIS software version 4.0.1 based on open data from the spatial analysis laboratory of the National Observatory for Spatial Planning).

This cross-sectional study was conducted from 15 April 2025 to 15 June 2025, using a self-administered questionnaire. We carried out a multicenter study in the cities of Kisangani, Bunia, Buta and Isiro, which are the capitals of Tshopo, Ituri, Bas-Uele and Haut-Uele provinces, respectively (Figure 1). These four cities were deliberately selected based on their geographical proximity and accessibility to the research team.

All public healthcare facilities likely to provide screening services were selected, including university hospitals, provincial hospitals, and GRHs. In addition, all private hospitals with technical facilities comparable to those of GRHs were included, particularly in Buta, in Bunia, and in Isiro, to enhance the representativeness of the selected cities.

The study was conducted in 17 eligible public and private healthcare facilities identified from provincial health division records across the four cities. Seven of these facilities are located in Kisangani/Tshopo province, including the University Clinics of Kisangani, Hôpital du Cinquantenaire de Kisangani, and all five GRHs: Makiso-Kisangani, Tshopo, Mangobo, Lubunga and Kabondo. The remaining cities included four facilities in Bunia, Ituri Province (University Clinics of Bunia, Bunia GRH, Evangelical Medical Center of Bunia, and Rwankole Hospital Center); three in Isiro, Haut-Uele province (University Clinics of Uele, Isiro GRH and Mayogo Hospital Center); and three in Buta, Bas-Uele province (Buta GRH, Bon Berger Center and Rubi Lusanga Center).

### 2.2. Study Population

The study population included 259 physicians employed in the 17 selected healthcare facilities. Eighty-four physicians were not included. This refers to 9 pediatricians, 12 internists, and 63 university hospital physicians practicing in departments other than obstetrics and gynecology (surgery, pediatrics, neuropsychiatry, and internal medicine).

### 2.3. Study Participants and Sampling

Initially, 175 physicians were eligible for the study: 32 from university hospitals and 143 from non-university hospitals. Eligible participants were physicians whose clinical responsibilities included the opportunity to perform CC screening. In university hospitals, this included physicians assigned to gynecology and obstetrics departments. Given the shortage of obstetrician–gynecologists, particularly in non-university hospitals, general practitioners and surgeons were also included because they may be involved in CC screening. This approach is consistent with the WHO-recommended “screen-and-treat” strategy, which promotes the decentralization of CC screening services in LMICs [22], where physicians often assume broader clinical responsibilities including CC screening [23].

Participants had to be at their workplaces at the time of the survey and provide their free and informed written consent.

**Figure 2:**
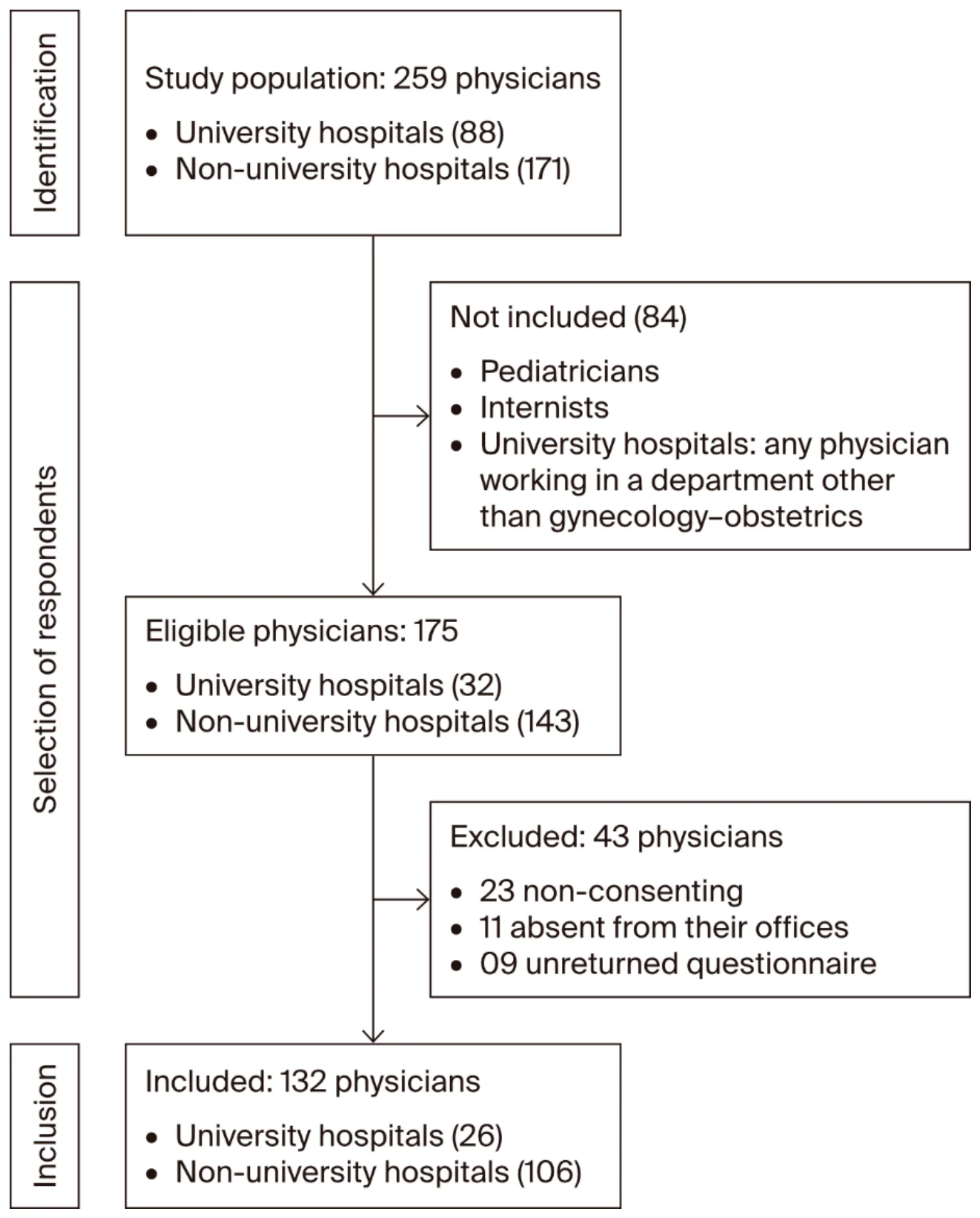
Selection of respondents.

We excluded 43 eligible physicians: 23 did not consent, 9 did not return the questionnaire, and 11 were absent at the time of the survey (physicians who were on leave or seconded outside their respective hospitals). The final sample comprised 132 physicians, including 26 physicians from university hospitals and 106 from non-university hospitals. Data were collected over six days in each city, with fieldwork conducted on three days per week (Monday, Wednesday, and Friday). Figure 2 illustrates the participant selection procedure.

### 2.4. Data Collection and Procedures

Data were collected using a structured, self-administered questionnaire developed based on item banks from African CC screening surveys [15,24], the available relevant literature [11,25], and WHO guidelines [7,26]. Some items assessing physicians’ self-reported clinical practices were adapted from the French Colposcopy Charter [27], as no comparable regional guidelines were available at the time of the study.

To evaluate the clarity and feasibility of the instrument, the French-language questionnaire was pretested with 15 physicians who were not included in the final study sample. However, no formal assessment of its content validity or reliability was performed. Prior to data collection, the study objectives and procedures were explained to each eligible physician. Physicians were approached individually in their offices and invited to participate voluntarily. Only those who provided written informed consent received the questionnaire and were included in the study. Each physician completed the questionnaire independently. The investigator was present to introduce the study and address any procedural questions.

The questionnaire comprised two main sections, assessing (i) knowledge and (ii) clinical practices regarding CC screening. A final section collected the sociodemographic and professional characteristics of the participants.

#### 2.4.1. Knowledge

The respondents were asked about the clinical symptoms of CC, the age range of women eligible for screening, risk factors, recommended intervals for cytology and HPV testing, available screening methods, and the test used to confirm the diagnosis. The symptoms considered correct were induced and/or postmenopausal metrorrhagia and malodorous leukorrhea. However, the overall assessment of physicians’ knowledge did not include knowledge of symptoms suggestive of CC or the use of biopsy for diagnostic confirmation.

The age groups of 25 to 65 years and 30 to 49 years were considered correct, based on the DRC’s 2015 national strategy [11] and WHO recommendations, which prioritize women aged 30–49 in LMICs [28].

Knowledge of CC risk factors was assessed based on the identification of nine established factors: early sexual intercourse, multiple sexual partners, parity over 5, first birth before the age of 20, repeated genital infections, especially with oncogenic HPV, low socioeconomic status, smoking, long-term oral contraception, and immunosuppression [25,29].

We considered the following cytology screening intervals appropriate: routine screening every three years or two cervical smears performed 12 months apart during the first year, followed by screening every three years [6,25]. We assessed respondents’ knowledge of the four CC screening methods: HPV testing, cytology, visual inspection with acetic acid (VIA) and visual inspection with Lugol’s iodine (VILI).

For each question, one or more points were awarded for correct answers, whereas zero points were assigned for incorrect or missing answers. We assigned a total of four points for the appropriate age group, five points for knowledge of risk factors, two points for the recommended cytology screening interval, two points for the recommended HPV testing interval, and four points for knowledge of screening methods. The overall score obtained by adding up the sub-ratings was 17. Overall knowledge was considered to be sufficient when the total score ranged from 8.5 to 17 and insufficient for a total score ranging from 0 to 8.

#### 2.4.2. Self-Reported Clinical Practices

Physicians’ clinical practices were evaluated using four criteria: (1) participation in continuing education, defined as either the successful completion of a certified colposcopy training program or professional internship in a CC screening service; (2) the number of screenings performed in the previous 12 months; (3) the methods used to screen; and (4) the number of patients treated for precancerous cervical lesions during the previous 12 months.

Self-reported clinical practices were assessed using a composite score ranging from 0 to 14 points. Continuing education contributed up to 3 points, with 3 points assigned to physicians who had completed either a certified colposcopy training program or an internship and 0 points to those who had completed neither. The number of women screened during the previous 12 months contributed up to 3 points: 0 points for no screening, 1 point for 1–24 screenings, 2 points for 25–49 screenings, and 3 points for ≥ 50 screenings.

Screening methods were scored out of 5 points: 1 point each for naked-eye VIA, naked-eye VILI, and cytology, and 2 points for colposcopy. Management of precancerous cervical lesions contributed up to 3 points, regardless of the treatment modality used (conization, cryotherapy and loop electrosurgical excision procedure [LEEP]): 0 points for no patients treated, 1 point for 1–14 patients, 2 points for 15–29 patients, and 3 points for ≥ 30 patients.

Overall practice was classified as low for a total score ranging from 0 to 6 and high for a total score ranging from 7 to 14. The survey questionnaire and the scoring system used are provided in Supplementary Materials (Supplementary File S2, Table S1).

#### 2.4.3. Operational Definition of Variables

Hospitals were grouped into university and non-university hospitals (provincial hospitals, GRHs and selected private hospitals). For the purposes of statistical analysis, physicians were grouped into two categories according to their specialties: obstetrician–gynecologists and others. The ‘others’ category includes general practitioners and surgeons—notably, those in non-university hospitals.

### 2.5. Statistical Analysis

The collected data were compiled in Excel^®^ 2016 and analyzed using R software version 4.6.0. Qualitative variables were summarized as frequencies (n) and percentages (%), while quantitative variables were expressed as means with standard deviations (SD) for normally distributed data or medians with interquartile ranges (IQR), otherwise. Following the study conducted in Brazzaville by Tebeu et al. [15], composite scores for knowledge and self-reported practices were dichotomized using a 50% threshold. Response categories in multiple-response questions were analyzed as separate binary variables.

Univariate analyses were performed to assess factors associated with physicians’ knowledge (insufficient vs. sufficient) and self-reported clinical practices (low vs. high). Associations were assessed using the Chi-square test or Fisher’s exact test, as appropriate. For the knowledge outcome, variables with a *p*-value ≤ 0.20 in univariate analyses were selected for inclusion in the initial multivariable logistic regression model. The initial model included gender, professional experience, physician’s specialty, location, and hospital profile. Multicollinearity was assessed using the variance inflation factor (VIF), with a threshold of 5; all candidate variables had VIF values < 5. A forward stepwise procedure based on the Akaike Information Criterion (AIC) was then applied to identify the most parsimonious model. The final model retained gender, location, and hospital profile. Multivariable analysis was not performed for self-reported practices because only six physicians had a high level of practice.

Associations were expressed as crude odds ratios (CORs) with 95% confidence intervals (95% CIs) for univariate analyses and as adjusted odds ratios (AORs) with 95% CIs for the final multivariable model. A two-sided *p*-value < 0.05 was considered statistically significant.

## 3. Results

### 3.1. Characteristics of Respondents

Table 1 shows that most participants (in university and non-university hospitals) were men (80.8% and 79.2%, respectively), with between 1 and 10 years of seniority (50.0% and 86.8%, respectively). All physicians interviewed at the university hospitals resided in either Kisangani, Tshopo province (80.8%), or Isiro, Haut-Uele province (19.2%). University hospitals had a higher proportion of obstetrician–gynecologists.

### 3.2. Respondent Knowledge Regarding Screening Modalities

Table 2 reveals that physicians working in non-university hospitals were significantly more likely to be unaware of the target age group for CC screening (*p*-value = 0.001). The screening methods most frequently listed by physicians in both university and non-university hospitals were cytology (57.7% vs. 39.6%) and VILI (46.2% vs. 40.6%). No physician responded to the question about the recommended interval for HPV testing. Physicians at university hospitals appeared to have better knowledge of the test used to confirm the diagnosis of cervical precancerous or cancerous lesions—namely, biopsy (*p*-value = 0.03).

### 3.3. Factors Associated with Knowledge of Cervical Cancer Screening

The overall knowledge score ranged from 0 to 14 out of a maximum possible score of 17, with a median of 5 (IQR: 3–8.5). Using the 50% threshold (8.5 out of 17 points), 25.8% of surveyed physicians had sufficient knowledge of CC screening. In the multivariable analysis, residence in Bunia (AOR: 4.71, 1.43–18.7; *p*-value = 0.02) and affiliation with a university hospital (AOR: 9.12, 2.57–38.9; *p*-value = 0.001) appeared to be significantly associated with sufficient knowledge (Table 3).

### 3.4. Physicians’ Self-Reported Cervical Cancer Screening and Management Activities

Table 4 presents respondents’ self-reported screening and treatment activities. A total of 57.7% of physicians in university hospitals and 49.0% of those in non-university hospitals reported screening 1–24 women in the previous 12 months. Physicians at university hospitals were more likely to report using appropriate screening methods than those at non-university hospitals (76.9% vs. 40.6%, *p*-value < 0.01). Naked-eye VILI was the most commonly reported screening method (57.7% vs. 32.1%, *p*-value = 0.03). Management of cervical precancerous lesions was not provided by the majority of surveyed physicians at either university hospitals (92.3%) or non-university hospitals (83.0%).

### 3.5. Factors Associated with Self-Reported Clinical Practices Among Physicians

The overall self-reported practice score ranged from 0 to 10 out of a maximum possible score of 14, with a median of 2 (IQR: 1–3). Using the threshold of 50% (7 out of 14 points), we found that 6 of 132 participants (4.5%) had a high level of screening- and management-related activities (Table 5). In our analysis, specialization in gynecology–obstetrics was the only factor significantly associated with high practice (COR: 8.46; CI: 1.47–71.7; *p*-value = 0.02).

## 4. Discussion

This study evaluates physicians’ knowledge of CC screening modalities and their self-reported clinical practices in northeastern DRC, while accounting for participants’ characteristics. However, the effects of hospital type, physician specialty, and physician location cannot be interpreted independently of one another because university hospitals include a much higher proportion of obstetrician–gynecologists; moreover, respondents from university hospitals were concentrated in two of the four cities that comprised our study setting (Table 1).

### 4.1. Respondents’ Knowledge of Cervical Cancer Screening Modalities

Our study showed that physicians working at university hospitals had better knowledge of the target age group for cervical cancer screening and the test used to confirm the diagnosis. HPV testing, which is currently recommended as the preferred primary screening method, was mentioned by only a minority of physicians (Table 2).

Since 2020, the WHO has recommended HPV testing as the preferred primary screening test for women starting at age 30, to be performed every five to ten years in the general population [6,26]. Further testing may be used for the triage process following a positive HPV test, as part of the “screen, triage and treat” approach [30,31].

While most LMICs do not have official guidelines [17,32], developed countries are updating their screening policies, notably France [33] and, more recently, Belgium [31]. According to the new recommendations from Sciensano (Belgium), cytology screening remains recommended every three years as the primary CC screening method for women aged 25 to 29 years. Between the ages of 30 and 64, it is replaced by an HPV test every five years, with reflex cytology in the event of a positive test. After age 64, a single screening or exit test may be performed [21,31].

The age groups considered appropriate for CC screening in our survey (30–49 and 25–65 years) [11,28] differ from the current target age range of 25–64 years recommended in European guidelines [21,31], which varies according to the screening method used (cytology or HPV testing). This discrepancy highlights the need to adapt WHO recommendations to the local context, while also reflecting the evolving nature of CC screening guidelines that physicians must navigate.

In our cohort, the limited knowledge and use of HPV testing observed among respondents may be partly explained by its limited availability and the lack of national screening guidelines, which contrasts with the situation in high-income countries [7,34,35].

Most physicians at non-university hospitals cited fewer than five of the nine risk factors (Table 2). Other African researchers have investigated the risk factors of CC separately. Having multiple sexual partners was the most frequently cited risk factor (83.2.% of participants) in Getahun et al.’s study in Ethiopia [24], while early sexual intercourse (45.5%) and smoking (21.5%) were the two most frequently mentioned risk factors reported by Marie Tebeu et al. in the study conducted in Cameroon [36]. These findings suggest that physicians’ knowledge of CC risk factors tends to focus on sexual-behavior-related factors, while other well-established determinants remain underrecognized.

We found that approximately one-quarter of physicians had sufficient knowledge (Table 3). Our results are similar to those of Ndizeye et al., who also observed that a minority of respondents (8.4%) had adequate knowledge in Burundi [23]. In addition, a recent meta-analysis reported that 49.68% (95% CI: 33.18–66.17%) of healthcare providers in SSA had good knowledge status [37]. Differences in proportions observed across studies may be partly explained by variations in the evaluation criteria used by the authors.

In our cohort, being affiliated with a university hospital and residing in Bunia, in Ituri Province, contributed to achieving a sufficient level of knowledge. However, in a study conducted in Congo-Brazzaville, Tebeu et al. found that the level of knowledge was significantly influenced by the respondent’s age and their seniority [15]. The association observed between residence in Bunia/Ituri Province and higher knowledge levels may be partially explained by its geographical proximity to Uganda, a country that has made measurable progress in cancer control and CC prevention programs [38]. This proximity may facilitate cross-border exchanges, access to training opportunities, and exposure to updated information and practices among physicians practicing in Bunia; however, this hypothesis warrants further investigation.

The knowledge gaps identified in our cohort may be partly attributable to the scarcity of local expertise and continuing education on the screening of gynecological cancers in the DRC. Several studies conducted in SSA confirm that insufficient knowledge among practitioners, as well as a lack of screening programs and guidelines, are the main factors contributing to the high incidence and mortality rates in LMICs [32,34,37].

Our results (Table 3) suggest that the involvement of university hospitals and skilled obstetrician–gynecologists in raising awareness among other healthcare professionals in the northeast of the DRC might represent a plausible avenue for improving their knowledge. These findings also highlight the value of establishing knowledge-sharing initiatives with external experts within or beyond academia and fostering collaboration within LMICs [39]. However, the effects of these interventions were not directly assessed in the present study.

### 4.2. Self-Reported Clinical Practices of Respondents

Most respondents had screened between 1 and 24 women in the previous 12 months, using VILI; the management of women with precancerous cervical lesions was also uncommon in both university and non-university hospitals (Table 4).

These findings are aligned with the statements that screening coverage remains suboptimal in SSA, apart from Southern Africa. Indeed, according to Yang et al., the proportion of women who had undergone at least two lifetime screening episodes by age 45 was estimated at 12% (95% CI: 11–18%) in 2020 [40].

Recommendations for screening may vary from country to country and from one region to another around the world [34]. Thus, in some Central African countries, particularly Rwanda and Cameroon, primary screening with VILI and/or VIA has been introduced [41,42]. These context-specific approaches are consistent with the screening practices reported by physicians in our study, where naked-eye VILI was the most frequently used screening method, reflecting both regional policies and resource constraints.

Effective treatment of precancerous and cancerous cervical lesions, as described in the WHO’s 90-70-90 strategy [6], requires that healthcare providers be proficient in using the various tools available. After the diagnosis is confirmed, some women can be eligible for ablative treatment (cryotherapy, loop electrosurgical excision or thermal ablation). Other patients may require surgical excision using a LEEP procedure or conization using a cold knife or an electric scalpel [30,43]. The use of these various tools has significantly reduced CC mortality in developed countries. To address the lack of local expertise, logistical constraints, and under-resourced healthcare facilities in LMICs [38,44], the WHO encourages ablative treatment options such as thermal ablation and cryotherapy for eligible women with cervical precancerous lesions [43,45].

The low level of therapeutic intervention in our cohort may be partly attributable to limited screening activity, as inadequate screening coverage inevitably reduces the identification of precancerous lesions eligible for timely treatment. Low treatment activity may also reflect referral patterns, a lack of equipment, restrictions in the scope of practice, a shortage of trained personnel, a limited number of obstetrician–gynecologists, and incomplete service pathways. These constraints collectively hinder the effective implementation of CC screening and management services.

In our sample, the treatment was performed by a limited number of obstetrician–gynecologists. The procedure often consisted of cold knife conization or using an electric scalpel (Table 4). Karim et al. had already reported limited training in gynecologic oncology in LMICs [44]. These observations underscore the need to strengthen and update obstetrics and gynecology training in the DRC and other LMICs, particularly through the integration of gynecologic oncology subspecialty training and continuing professional development programs [39,46]. Enhancing clinicians’ capacity to manage precancerous lesions is essential to ensure the effectiveness of CC screening programs and to reduce progression to invasive disease.

In Central Africa in general, and in the DRC in particular, much effort is still needed to ensure that hospitals provide quality care during CC screening. In 2023, only 37% of level III health facilities in Uganda were adequately equipped to screen for and manage precancerous cervical lesions [38]. Studies conducted in Cameroon have also identified barriers related to the quality of healthcare services [47,48].

Consistent with these findings, we found that only 4.5% of participants had high self-reported practices (Table 5) and obstetrician–gynecologists seem more likely to have higher practices than others (*p*-value = 0.02). However, these indicators may reflect institutional resources, equipment availability, patient flow, and scope of practice rather than individual physician practice quality. Some neighboring countries appear to have made better progress in preventing CC. Notably, 60% of Health Centers with grades IV and above in Gulu/Uganda had adequate resources for prevention [38].

Since 2017, the WHO [49] has identified numerous challenges relating to the prevention and control of CC in Africa, including insufficient information and skills, a lack of secondary prevention measures (screening), the inaccessibility of tertiary measures (treatment), and a lack of guidelines. Indeed, in 2020, approximately 30% of LMICs reported that pathology services, cancer surgery, chemotherapy and radiotherapy were generally available in public hospitals, compared with over 90% of high-income countries [50]. Four years later, Kassa et al. [51] conducted an analysis indicating that a failure to implement an oncology prevention policy, a lack of qualified personnel, and scarcity of resources remain obstacles that must be overcome in order to improve CC screening services in LMICs. The meta-analysis published by Delie et al. in 2024 revealed that only 17.23% (95% CI: 6.08–28.37%) of healthcare providers in SSA had ever screened for CC [37].

In light of our findings, strengthening the practical skills of physicians in both university and non-university hospitals in cervical cancer screening appears essential. This may be achieved through targeted mentorship, while leveraging the experience of gynecologists with more established screening practices.

### 4.3. Strengths and Limitations of the Study

A major strength of this study is that it represents one of the first assessments of the current status of CC screening based on a survey of healthcare professionals in a largely unexplored region of the DRC. In addition, the findings identify potential avenues for improving CC screening services in northeastern DRC and may inform future research, guide targeted interventions, and support the development of context-appropriate CC screening strategies in other regions of the country.

However, our study has some limitations:

- First, the assessment of clinical practices relied on self-reported data, which may be affected by recall and social desirability biases. Second, although the questionnaire was pretested, it was not formally validated. Some topics may not have been addressed in sufficient depth, potentially limiting the comprehensiveness of the assessment and resulting in disproportionate weighting of individual items. In addition, clustering of responses among physicians practicing at the same facility may have affected the precision of the estimates.
- Third, the geographic distribution of university hospitals was uneven across the four study cities: no university hospital was available in Buta (Bas-Uele province), while five physicians from the newly established university hospital in Bunia (Ituri province) did not consent to participate. This highlights disparities in the availability of university hospitals and obstetrician–gynecologists across northeastern DRC. The non-probability sampling approach and non-participation of 43 eligible physicians may have introduced selection bias.
- Fourth, the small number of physicians with sufficient knowledge or a high level of self-reported practices limited the scope of the analyses, resulting in wide confidence intervals and reduced statistical power. This limitation was compounded by the disproportionate concentration of obstetrician–gynecologists in university hospitals. Therefore, the observed association between hospital type and the study outcomes should be interpreted with caution, as it is closely intertwined with physicians’ specialties and geographic location.
- Finally, since this study was conducted in the provincial capitals of four of the DRC’s 26 provinces, our findings may not be generalizable nationwide, even though some realities may be common across the DRC.

Future studies encompassing a wider geographical coverage of the DRC are needed to provide a more comprehensive understanding of physicians’ knowledge and clinical practices regarding CC prevention and screening at the national level. Nevertheless, our findings clearly underscore the urgent need to strengthen the capacity of healthcare providers to improve CC prevention and screening in the DRC.

## 5. Conclusions

Within the bounds of this cross-sectional survey conducted in selected hospitals across four provincial capitals, only a small proportion of physicians had sufficient knowledge of the CC screening modalities and/or reported high levels of clinical practice. Although estimates varied across professional and institutional groups, clear differences were difficult to disentangle due to the sample structure. Our findings suggest that improving the screening and management of precancerous cervical lesions in clinical practice may require mentorship from experienced obstetrician–gynecologists and greater collaboration between physicians from university and non-university hospitals, both within and across provinces. Future research should jointly measure physician knowledge, facility capacity, referral pathways, and patient-level service delivery in a wider geographic area of the DRC.

## Figures and Tables

**Table 1:** Study participants’ characteristics.

Characteristics of Respondents	Non-University Hospitals N = 106 n (%)	University Hospitals N = 26 n (%)
**Gender**		
Female	22 (20.8)	5 (19.2)
Male	84 (79.2)	21 (80.8)
**Professional experience**		
1 to 10 years	92 (86.8)	13 (50.0)
Over 10 years	14 (13.2)	13 (50.0)
**Physician’s specialty**		
Others	102 (96.2)	3 (11.5)
Obstetrician–gynecologists	4 (3.8)	23 (88.5)
**Residence (city/province)**		
Buta/Bas-Uele	18 (17.0)	0 (0.0)
Isiro/Haut-Uele	19 (17.9)	5 (19.2)
Bunia/Ituri	36 (34.0)	0 (0.0)
Kisangani/Tshopo	33 (31.1)	21 (80.8)

**Table 2:** Knowledge of cervical cancer screening modalities among physicians.

Knowledge of Respondents	Non-University Hospitals N = 106 n (%)	University Hospitals N = 26 n (%)	*p*-Value
**Warning signs suggestive of cervical cancer**			
Induced metrorrhagia	40 (37.7)	14 (53.8)	0.13
Smelly leucorrhea	13 (12.3)	4 (15.4)	0.7
Incorrect symptoms	62 (58.5)	12 (46.2)	0.3
**Target age group**			
30 to 49 years	34 (32.1)	14 (53.8)	0.039
25 to 65 years	3 (2.8)	4 (15.4)	0.028
Incorrect	69 (65.1)	8 (30.8)	0.001
**Number of risk factors cited**			
1 to 4	63 (59.4)	12 (46.2)	0.22
5 to 9	43 (40.6)	14 (53.8)	0.22
**Recommended cytology screening interval**			
Twice in 1 year, then every 3 years	4 (3.8)	4 (15.4)	0.058
Every 3 years	13 (12.3)	4 (15.4)	0.7
Incorrect answer	89 (84.0)	18 (69.2)	0.1
**Screening methods cited**			
Cytology	42 (39.6)	15 (57.7)	0.15
HPV Test	14 (13.2)	2 (7.7)	0.74
VILI	43 (40.6)	12 (46.2)	0.77
VIA	37 (34.9)	12 (46.2)	0.40
Incorrect	37 (34.9)	12 (46.2)	0.40
**Test used to confirm the diagnosis**			
Biopsy	82 (77.4)	25 (96.2)	0.03
Colposcopy	43 (40.6)	12 (46.2)	0.77
Others: VILI, VIA. and Pap test	25 (23.6)	1 (3.8)	0.03

*p*-values are unadjusted for multiple testing; comparisons are exploratory.

**Table 3:** Association between physicians’ characteristics and knowledge of cervical cancer screening.

Characteristics of Respondents	Insufficient Knowledge	Sufficient Knowledge	COR	*p*-Value	AOR	*p*-Value
	n = 98 (74.2%)	n = 34 (25.8%)				
**Gender**						
Female	25 (92.6%)	2 (7.4%)	Ref.	Ref.	—	
Male	73 (69.5%)	32 (30.5%)	5.10 [1.39—35.8]	0.01	3.57 [0.83–25.00]	0.12
**Professional experience**						
≤10 years	81 (77.1%)	24 (22.9%)	Ref.	Ref.		
>10 years	17 (63.0%)	10 (37.0%)	1.98 [0,78—4.90]	0.15		
**Physician’s specialty**						
Others	83 (79.0%)	22 (21.0%)	Ref.	Ref.		
Obstetrician–gynecologists	15 (55.6%)	12 (44.4%)	2.99 [1.20—7.40]	0.02		
**Residence (City/Province)**						
Kisangani/Tshopo	39 (72.2%)	15 (27.8%)	Ref.	Ref.	—	
Buta/Bas Uele	14 (77.8%)	4 (22.2%)	0.76 [0.19—2.57]	0.67	1.88 [0.39–9.21]	0.40
Isiro/Haut Uele	23 (95.8%)	1 (4.2%)	0.13 [0.01—0.71]	0.01	0.17 [0.01–1.15]	0.12
Ituri/Bunia	22 (61.1%)	14 (38.9%)	1.64 [0.66—4.09]	0.28	4.71 [1.43–18.7]	0.02
**Hospital Profile**						
Non-university hospitals	84 (79.2%)	22 (20.8%)	Ref.	Ref.	—	—
University hospitals	14 (53.8%)	12 (46.2%)	3.24 [1.29—8.11]	0.01	9.12 [2.57–38.9]	0.001

**Table 4:** Activities related to the screening and management of precancerous cervical lesions.

Self-Reported Screening and Management Activities	Non-University Hospitals N = 106 n (%)	University Hospitals N = 26 n (%)	*p*-Value
**Continuing education on cervical cancer screening**			1.00
Completed	5 (4.7)	1 (3.8)	
Not completed	101 (95.3)	25 (96.2)	
**Women screened within the last 12 months**			
0	41 (38.7)	9 (34.6)	0.88
1 to 24	52 (49.0)	15 (57.7)	0.57
25 to 49	9 (8.5)	2 (7.7)	1.00
≥50	4 (3.8)	0 (0.0)	0.58
**Screening methods used in clinical practice**			
**Appropriate method**	43 (40.6)	20 (76.9)	<0.01
Naked-eye VILI	34 (32.1)	15 (57.7)	0.03
Naked-eye VIA	12 (11.3)	5 (19.2)	0.33
Cytology (Pap test)	13 (12.3)	6 (23.1)	0.21
Colposcopy (VILI and VIA)	3 (2.8)	0 (0.0)	1.00
**Inappropriate method (speculum examination, vaginal swab)**	33 (31.1)	4 (15.4)	0.17
**None**	30 (28.3)	2 (7.7)	0.05
**Treatment modalities used in clinical practice**			
Conization (cold knife, electrical scalpels)	12 (11.3)	2 (7.7)	0.74
Loop Electrosurgical Excision Procedure	4 (3.8)	0 (0.0)	0.58
Cryotherapy	3 (2.8)	0 (0.0)	1.00
None	88 (83.0)	24 (92.3)	0.36
**Number of patients treated in the previous 12 months**			
0	88 (83.0)	24 (92.3)	0.36
1 to 14	13 (12.3)	2 (7.7)	0.73
15 to 29	1 (0.9)	0 (0.0)	1.00
≥30	4 (3.8)	0 (0.0)	0.58

*p*-values are unadjusted for multiple testing; comparisons are exploratory.

**Table 5:** Association between physicians’ characteristics and their self-reported clinical practices.

Physicians’ Characteristics	Total	Low Practice	High Practice	COR	*p*-Value
	N (%)	n = 126 (95.5%)	n = 6 (4.5%)		
**Gender**					
Female	27 (20.5%)	26 (96.3%)	1 (3.7%)	Ref.	Ref.
Male	105 (79.5%)	100 (95.2%)	5 (4.8%)	1.17 [0.17—32.1]	0.89
**Professional experience (years)**					
≤10	105 (79.5%)	100 (95.2%)	5 (4.8%)	Ref.	Ref.
>10	27 (20.5%)	26 (96.3%)	1 (3.7%)	0.85 [0.03—5.87]	0.89
**Physician’s specialty**					
Others	105 (79.5%)	103 (98.1%)	2 (1.9%)	Ref.	Ref.
Obstetrician–gynecologists	27 (20.5%)	23 (85.2%)	4 (14.8%)	8.46 [1.47—71.7]	0.02
**Residence (city/province)**					
Kisangani/Tshopo	54 (40.9%)	52 (96.3%)	2 (3.7%)	Ref.	Ref.
Buta/Bas Uele	18 (13.6%)	18 (100.0%)	0 (0.0%)	NA	NA
Isiro/Haut Uele	24 (18.2%)	24 (100.0%)	0 (0.0%)	NA	NA
Ituri/Bunia	36 (27.3%)	32 (88.9%)	4 (11.1%)	NA	NA
**Hospital profile**					
Non-university	106 (80.3%)	100 (94.3%)	6 (5.7%)	Ref.	Ref.
University	26 (19.7%)	26 (100.0%)	0 (0.0%)	NA	NA
**Level of knowledge**					
Insufficient	98 (74.2%)	95 (96.9%)	3 (3.1%)	Ref.	Ref.
Sufficient	34 (25.8%)	31 (91.2%)	3 (8.8%)	3.03 [0.50—18.5]	0.22

## Data Availability

The datasets generated and/or analyzed during the study are not publicly available due to privacy and ethical restrictions protecting the confidentiality of study participants. However, anonymized data may be made available from the corresponding author upon reasonable request and subject to approval by the School of Public Health at the University of Kinshasa ethics committee.

## Supplementary Materials

The following supporting information can be downloaded at https://www.mdpi.com/article/10.3390/healthcare14213594/s1, Supplementary File S1, Informed consent form. Supplementary File S2, Survey questionnaire. Table S1, Scoring and rating of knowledge and self-reported practices.
