# Supplementary material for: Assessment of Physicians’ Knowledge and Practices Regarding Cervical Cancer Screening in University and Non-University Hospitals in Northeastern Democratic Republic of the Congo
Source: Healthcare (Basel). 2026 Oct 23;14(21):3594. doi: 10.3390/healthcare14213594 (PMC13763185; doi:10.3390/healthcare14213594)
Supplement: Supplementary file 1 [file healthcare-14-03594-s001.zip › Supplementary File S1.pdf]

Fichier supplémentaire S1. **FORMULAIRE DE CONSENTEMENT ECLAIRE**

Intitulé de l'étude :

Connaissance et pratique des médecins sur le dépistage du cancer du col dans les hôpitaux universitaires et non-universitaires au nord-est de la RDC

Partie pour le/la participant (e)

Je certifie avoir accepté librement et volontairement de participer à cette étude portant sur les connaissances et les pratiques professionnelles des médecins relatives au dépistage du cancer du col de l'utérus. Cette participation consiste à répondre à un questionnaire portant sur mes connaissances théoriques et mes pratiques cliniques.

J'ai reçu une fiche d'information écrite ainsi que les explications nécessaires de la part de l'investigateur. J'ai pu poser toutes les questions que je souhaitais et j'ai obtenu des réponses satisfaisantes. Je comprends que ma participation est volontaire et que j'ai le droit de me retirer de l'étude à tout moment, sans que cela ne me porte préjudice.

J'autorise l'utilisation, l'analyse et la publication de mes données sous une forme anonymisée, dans le respect de la confidentialité, des principes éthiques notamment les principes de la déclaration d'Helsinki ; et de la réglementation relative à la protection des données personnelles.

Date :……………/………………/………………………………

Signature :……………………………………………………….

A compléter par la personne qui recueille le consentement

Je soussignée**…………………………………..**certifie avoir informé la /le participant(e) de tous les aspects pertinents de cette étude. Je certifie qu'elle a volontairement accepté de participer à l'étude.

Date : …………/…………/………………………
Signature :………………………………………..
E-mail : dr.yneema@gmail.com
Institution : Cliniques Universitaires de Kisangani, République Démocratique du Congo

Supplementary File S1-ENG **. INFORMED CONSENT FORM**

Study Title:

"Assessment of physicians 'knowledge and practices regarding cervical cancer screening in university and non-university hospitals in northeastern Democratic Republic of the Congo "

Participant Section

I certify that I have freely and voluntarily agreed to participate in this study on physicians' knowledge and clinical practices regarding cervical cancer screening by completing a questionnaire on my theoretical knowledge and professional practices.

I confirm that I have received a written information sheet and the necessary explanations from the investigator. I have had the opportunity to ask questions and received satisfactory answers. I understand that my participation is entirely voluntary and that I may withdraw from the study at any time without any adverse consequences.

I authorize the collection, analysis, and publication of my data in anonymized form, in accordance with ethical principles, the principles of the Declaration of Helsinki, confidentiality requirements, and the General Data Protection Regulation (GDPR).

Date: ………………/………………/…………………

Signature :…………………………………………….

To be completed by the person obtaining consent

I, the undersigned…………………………………..certify that I have informed the participant of all relevant aspects of this study. I certify that she has voluntarily agreed to participate in the study.

Date: ……………/………………/………………………
Signature:………………………………………………...
E-mail : dr.yneema@gmail.com
Institution : The Kisangani University Clinics, Democratic Republic of the Congo
