# Supplementary material for: Assessment of Physicians’ Knowledge and Practices Regarding Cervical Cancer Screening in University and Non-University Hospitals in Northeastern Democratic Republic of the Congo
Source: Healthcare (Basel). 2026 Oct 23;14(21):3594. doi: 10.3390/healthcare14213594 (PMC13763185; doi:10.3390/healthcare14213594)
Supplement: Supplementary file 1 [file healthcare-14-03594-s001.zip › Supplementary File S2.pdf]

Fichier supplémentaire S2**. <u>QUESTIONNAIRE D'ENQUETE</u>**

Thème : Évaluation des connaissances et des pratiques des médecins en matière de dépistage du cancer du col de l'utérus dans les hôpitaux universitaires et non universitaires, au nord-est de la République démocratique du Congo

Cher médecin,
Nous sollicitons votre disponibilité pour répondre aux questions ci-dessous. Vos réponses nous aideront à identifier les obstacles au dépistage du cancer du col de l'utérus dans le nord-est de la République démocratique du Congo, en évaluant les connaissances et les pratiques des médecins.

A. CONNAISSANCES

1. Quels sont les symptômes pouvant permettre de suspecter un cancer du col utérin au cours d'une consultation médicale?
   a. Metrorragie spontannée
   b. Metrorragie de contact
   c. Douleurs hypogastriques
   d. Leucorrhées sales ou malodorantes
   e. Autres symptômes à préciser……………………………………………….

2. Quelle est la tranche d'âge des femmes à cibler lors du dépistage du cancer du col utérin au sein de la population générale ?
   a. 30 à 49 ans
   b. 15 à 64 ans
   c. 25 to 65 ans
   d. Tout âge dès les ménarches
   e. Autre tranche d'âge à spécifier…………………………………

3. En ce qui concerne les tests de dépistage et la confirmation du diagnostic

3.1 Lister les examens auxquels on peut faire recours pour dépister les lésions précancéreuses du col utérin.

……………………………………………………………………………………………
……………………………………………………………………………………………
……………………………………………………………………………………………
……………………………………………………………………………………………

3.2 Quel examen peut être utilisé pour confirmer le diagnostic de certitude d'une lésion précancéreuse ou cancéreuse du col utérin?

a. La colposcopie
b. La biopsie
c. Autre examen (à préciser)…………………………………………………………….

4. Quel est l'intervalle recommandé entre les examens suivants dans le cadre du dépistage systématique du cancer du col de l'utérus dans la population générale ?

a. La cytologie cervicale

……………………………………………………………………………………………
……………………………………………………………………………………………
……………………………………………………………………………………………

b. Le test HPV

……………………………………………………………………………………………
……………………………………………………………………………………………

5. Pouvez-vous énumérer les facteurs de risque du cancer du col de l'utérus reconnus dans la littérature scientifique ?

……………………………………………………..……………………………………….
……………………………………………………………………………………………
……………………………………………………………………………………………

B. PRATIQUES CLINIQUES

6. Avez-vous suivi une formation spécifique (formation certifiante en colposcopie ou stage professionnel dans un service spécialisé) pour le dépistage et la prise en charge des lésions précancéreuses du col?

a. Oui
– Par quelle organization?……………………………………………………
– Dans quel pays (RDC ou un autre pays à préciser) ?

……………………………………………………………………………………….

b. Non, je n'ai reçu aucune formation spécifique .

7. Parmi les différentes modalités de dépistage listées ci-dessous, indiquez celle à laquelle vous recourez dans vos pratiques cliniques pour dépister le cancer du col de l'utérus.

a. Inspection visuelle à l'acide acétique (VIA)
b. Inspection visuelle au Lugol iodine (VILI)
c. Frottis en phase liquide (Thin Prep)

d. Frottis conventionnel
e. Colposcopie (avec VIA et VIL)
f. Autre (veuillez préciser) ………………………………………………………….

8. Pouvez-vous préciser le nombre de femmes que vous avez eu à dépister au cours des 12 derniers mois?...
9. Avez-vous l'habileté de traiter les femmes avec lésions précancéreuses du col?
a. Oui
b. Non

10. Si vous êtes en mesure de prendre en charge les femmes présentant des lésions précancéreuses du col de l'utérus ,
10.1 Combien de femmes présentant des lésions précancéreuses du col avez-vous traitées au cours des 12 derniers mois (effectif)…………………………………………….
10.2 Parmi les outils de traitement ci-après, quels sont les types de traitement que vous pouvez offrir pour soigner les lésions précancéreuses du col dans vos pratiques cliniques ?
a. Conisation (bistouris à froid, bistouris électrique)
b. Excision électrochirurgicale à l'anse (LEEP)
c. Cryothérapie
d. Thermocoagulation
e. Autre (à préciser)………………………………………………………………..

C. IDENTIFICATION DU MEDECIN ENQUETE

- Sexe……………………………………………………………………………………..
- Durée de l'expérience professionnelle (nombre d'année) …………………………..
- Spécialité du médecin……………………………………………………………………
- Nom de la structure sanitaire……………………………………………………………
- Localisation (Ville/ Province) : ………………………………………………………….
- Profil de la structure sanitaire (Hôpital Privé, Hôpital General de Référence ,Hôpital provincial , Cliniques Universitaires etc.) ………………………………….
- Date de remplissage du questionnaire :………………………………………………..

Merci d'avoir accepté consacré de votre temps pour répondre à notre questionnaire
Dr NEEMA UFOY MUNGU Yvette/dr.yneema@gmail.com (Investigatrice)

Supplementary File S2-ENG **: SURVEY QUESTIONNAIRE**

Assessment of physicians 'knowledge and practices regarding cervical cancer screening in university and non-university hospitals in northeastern Democratic Republic of the Congo

Dear Physician,
Please answer the questions below. Your responses will help us identify barriers to cervical cancer screening in northeastern Democratic Republic of the Congo by assessing physicians' knowledge and clinical practices.

A. KNOWLEDGE

1. What signs and symptoms may suggest cervical cancer during a medical consultation?
   a. Spontaneous metrorrhagia,
   b. Induced metrorrhagia
   c. Hypogastric pain
   d. Smelly leucorrhea
   e. Other symptoms to be specified…………………………………………….

2. What is the recommended age range for routine cervical cancer screening in the general population?
   a. 30 to 49 years old
   b. 15 to 64 years
   c. 25 to 65 years
   d. Any age, starting from menarche.
   e. Other age group (to be specified) ………………………………………

3. Regarding cervical cancer screening and diagnostic confirmation ,

3.1 List the tests that can be used to screen for cervical precancerous lesions
…………………………………………………………………………………
…………………………………………………………………………………
…………………………………………………………………………………
…………………………………………………………………………………

3.2 Which tests can be used to confirm the diagnosis of a cervical precancerous or cancerous lesion?
   a. Colposcopy
   b. Biopsy
   c. Other tests (please specify)…………………………………………………

4. What are the recommended intervals between the following screening tests for cervical cancer in the general population?
   a. Cervical cytology
      ………………………………………………………………………………
      ………………………………………………………………………………
      ………………………………………………………………………………
   b. Papillomavirus test (HPV testing)
      ………………………………………………………………………………
      ………………………………………………………………………………

5. Please list the risk factors for cervical cancer recognized in the scientific literature ?
………………………………………………………..……………………………………
…………………………………………………………………………………………
…………………………………………………………………………………………
…………………………………………………………………………………………..

B . CLINICAL PRACTICES

6. Have you received any specific training in cervical cancer screening and the management of cervical precancerous lesions (e.g., certified colposcopy training or professional internship in a cervical cancer screening service)?
   a. Yes
      – By which organization(name)?…………………………………………
      – Where (DRC or another country to be specified)?……………………
   b. No, I have not received any specific training

7. Please indicate which of the following screening methods you use in your clinical practice to screen for cervical cancer.
   a. Visual Inspection with Acetic acid (VIA)
   b. Visual Inspection with Lugol's Iodine (VILI)
   c. Liquid- phase smear (Thin Prep)
   d. Conventional smear (
   e. Colposcopy (with VIA and VIL)
   f. Other (to be specified) …………………………………………………..

8. How many women have you screened for cervical cancer in the past 12 months (specify the number)?…………………………………………………………

9. Do you provide treatment for cervical precancerous or cancerous lesions in your clinical practice?
   a. Yes
   b. No

10. If you are able to manage women with cervical precancerous lesions,

10.1 How many women with cervical precancerous lesions have you managed in the past 12 months (*number of women to be specified*)?..

10.2 What types of treatment for cervical precancerous lesions do you provide in your clinical practice?
   a. Conization (cold knife, electrical scalpel)
   b. Loop Electrosurgical Excision Procedure (LEEP)
   c. Cryotherapy
   d. Thermocoagulation
   e. Other (to be specified)…………………………………………………..

C. IDENTIFICATION OF THE SURVEYED PHYSICIAN

- Gender………………………………………………………………………….
- Professional experience (years)…………………………………………………
- Medical specialty……………………………………………………………..
- Name of the healthcare facility…………………………………………………
- City/Province : ………………………………………………………………
- Type of facility (Private hospital, General Referral Hospital, Regional hospital, University Clinic etc.) ……………………………………………………………
- Date ……………………………………………………………………………

Thank you for taking time to answer our questions.

Dr NEEMA UFOY MUNGU Yvette/dr.yneema@gmail.com
