# Supplementary material for: Assessment of Physicians’ Knowledge and Practices Regarding Cervical Cancer Screening in University and Non-University Hospitals in Northeastern Democratic Republic of the Congo
Source: Healthcare (Basel). 2026 Oct 23;14(21):3594. doi: 10.3390/healthcare14213594 (PMC13763185; doi:10.3390/healthcare14213594)
Supplement: Supplementary file 1 [file healthcare-14-03594-s001.zip › Table S1.pdf]

Table S1 .Scoring and Rating of Knowledge and Self-Reported Practices

№	Items	Response options	Scoring
	**Knowledge assessment**		**Maximum score:** 17
1	Target age group for routine cervical cancer screening in the general population	Correct age group: 25 to 65 years or 30 to 49 years	4
		Incorrect age group (0)	0
2	Listed risk factors of cervical cancer	Early sexual intercourse	0.5
		Multiple sexual partners	0.5
		Parity over 5	0.5
		First birth before the age of 20	0.5
		Repeated genital infections, especially with oncogenic HPV	1
		Low socioeconomic status	0.5
		Smoking	0.5
		Long-term oral contraception	0.5
		Immunosuppression	0.5
3	4.1 Recommended cervical cytology screening interval in the general population	Twice in 1 year, then every 3 years or every 3 years	2
		Incorrect answer	0
	4.2 recommended screening interval for HPV testing in the general population	Every 5 to 10 years	2
		No answer	0
4	Screening methods cited	Cytology	1
		HPV test	1
		VILI	1
		VIA	1
		Incorrect	0
		Colposcopy (incorrect)	0
		Others: VILI, VIA and Pap test (incorrect)	0
Overall Assessment of Knowledge<br>▪ Sufficient: score ranging from 0 to 8 and,<br>▪ Insufficient: score ranging from 8.5 to 17			

	**Self-reported practice assessment**		**Maximum score: 14**
1.	Continuing education on cervical cancer screening (certified colposcopy training or professional internship in a cervical cancer screening service)	Completed	3
		Not completed	0
2.	Women screened within the last 12 months	0	0
		1 to 24	1
		25 to 49	2
		≥ 50	3
3.	Screening methods used in clinical practice	Naked-eye VILI	1
		Naked-eye VIA	1
		Cytology	1
		Colposcopy (VIA and VILI)	2
		Inappropriate method (speculum examination, vaginal swab)	0
		None	0
4.	Number of patients treated in the previous 12 months	0	0
		0 to 14	1
		15 to 29	2
		≥ 30	3
Overall Assessment of self-reported Practices<br>▪ Low for a total score ranging from 0 to 6 and,<br>▪ High for a total score ranging from 7 to 14.			
